# A Comparison of Combustion Properties in Biomass–Coal Blends Using Characteristic and Kinetic Analyses

**DOI:** 10.3390/ijerph182412980

**Published:** 2021-12-09

**Authors:** Yalin Wang, Beibei Yan, Yu Wang, Jiahao Zhang, Xiaozhong Chen, Rob J. M. Bastiaans

**Affiliations:** 1 School of Environmental Science and Engineering, Tianjin University, Tianjin 300072, China; yanbeibei@tju.edu.cn (B.Y.); Carl@solidat.net (X.C.); 2 Department of Mechanical Engineering, Eindhoven University of Technology, P.O. Box 513, 5600 MB Eindhoven, The Netherlands; y.wang14@tue.nl (Y.W.); r.j.m.bastiaans@tue.nl (R.J.M.B.); 3 Key Laboratory of Efficient Utilization of Low and Medium Grade Energy, Ministry of Education, School of Mechanical Engineering, Tianjin University, Tianjin 300072, China; 4 Bahen Centre for Information Technology, Department of Computer Science, University of Toronto, 40 St. George Street, Toronto, ON M5S 2E4, Canada; jiahaoz.zhang@mail.utoronto.ca

**Keywords:** biomass–coal blends, combustion, thermogravimetric analysis (TGA), combustion properties, kinetic analysis, activation energy

## Abstract

This paper presents comparative research on the combustion of coal, wheat, corn straw (CS), beet residues after extracting sugar (BR), and their blends, coal–corn straw blends (CCSBs), coal–wheat blends (CWBs), and coal–beet residue blends (CBRBs), using thermogravimetric (TG) analysis under 10, 20, 30, 40 and 50 °C/min. The test results indicate that CS and wheat show better combustion properties than BR, which are recommended to be used in biomass combustion. Under the heating rate of 20 °C/min, the coal has the longest thermal reaction time when compared with 10 and 30 °C/min. Adding coal to the biomass can improve the burnout level of biomass materials (BM), reduce the burning speed, and make the reaction more thorough. The authors employed the Flynn–Wall–Ozawa (FWO) method and the Kissinger–Akahira–Sunose (KAS) method to calculate kinetics parameters. It was proven that overall, the FWO method is better than the KAS method for coal, BM, and coal–biomass blends (CBBs), as it provides higher correlations in this study. It is shown that adding coal to wheat and BR decreases the activation energy and makes conversion more stable under particular *α*. The authors selected a wider range of biomass raw materials, made more kinds of CBB, and conducted more studies on different heating rates. This research can provide useful insights into how to choose agricultural residuals and how to use them.

## 1. Introduction

Biomass is a sustainable energy source that is suitable for human needs. Biomass is an appropriate replacement option to decrease the CO_2_ emissions from nonrenewable fossil fuels in favor of sustainable and renewable energy sources [1]. Biomass can reduce CO_2_ in the course of lignocellulosic in the process of photosynthesis [1]. The CO_2_ discharged from the burning process of biomass materials (BMs) makes no net contribution to the greenhouse effect. Therefore, biomass is a prospective material in the near future to replace coal and other fossil fuels, e.g., by blending it with coal. 

Co-combustion of coal and biomass is one of the most prospective methods to use for renewable energy sources during the transition from nonrenewable fossil fuels. As coal is a vital energy source, it will be used for a long time [2]. In China, coal accounts for around 70% of the primary energy supply, which is much higher than the global average [3]. In India, coal is the source of more than 50% of commercial primary energy [4]. Co-combustion is a low-risk and low-cost approach [5]. Coal and biomass have different combustion processes. Compared with coal, biomass has a higher content of oxygen, moisture content, and volatile matter [6]. Biomass also has a different mineral matter composition, which can cause slagging and corrosion of the boilers [7]. Thus, it is very important to research characteristics of co-combustion of biomass and coal under different combustion conditions [8]. There are a few beneficial results of mixing biomass together with coal or with other kinds of fuels before burning. The mixture of biofuel products and coal can achieve better control of the whole combustion process [9], listed in the following three points: Firstly, the co-combustion will reduce the consumption of nonrenewable energy. Secondly, in co-combustion, biomass can provide a more stable flame, because of the higher content of volatile matter more than 35% [10]. Thirdly, the co-combustion of coal and biomass can decrease the issues of ash deposition and foul, compared with biomass combustion only [11]. Therefore, adding biomass to existing coal-fired plants will provide considerable benefits in economic, environmental, and technical areas [12]. 

Thermogravimetric analysis (TGA) is a technology that allows measuring the mass loss of a sample as a function of time and temperature [13]. TGA is more suitable for the small quantities of materials in the combustion area, compared with other analytical methods [14]. Many papers used different methods to describe thermal degradation via nonisothermal methods and TGA [15]. To obtain kinetic parameters, existing methods can be applied to the curves obtained from TGA, and the kinetics of the thermal reactions could be defined by using the Arrhenius equation. The separate slopes of constant mass loss can be obtained from TGA [9,16,17]. Combustion reactivity studies by TGA can be found in the literature for both coal [18,19,20] and biomass fuels [21,22,23], but there exist a few comparison studies [24,25] and even fewer related studies about coal–biomass blends (CBBs) [26]. However, it is not enough to research biomass combustion properties only, because biomass itself cannot produce enough power in the current society. Therefore, the combustion of CBB has received increasing attention among researchers. Magalhães et al. [27] conducted research on the combustion characteristics and kinetics of Turkish lignite–poppy capsule pulp blends. The results showed that there might a synergistic interaction between the poppy capsule pulp and the lignite samples under the atmosphere of CO_2_ and N_2_. Ullah et al. [28] illustrated that the thermal reactivity and ignition performance of coal were upgraded by adding pinewood via TGA. Wang et al. [29] observed that the ignition property was enhanced by adding more biomass into the coal during the co-combustion procedure. Chansa et al. [30] used the Coats–Redfern method to evaluate the kinetic parameters of biomass, coal, and CBB under oxyfuel atmosphere. 

Thus far, the combustion of CBB has received considerable attention. However, it still needs further research in areas such as the comparison of combustion performance between different kinds of CBB to investigate how different biomass types affect coal’s ignition and burnout activity during co-combustion. This is also the main purpose of this study. Therefore, seven materials were used in this paper, as already mentioned in the abstract. These are coal, wheat, corn straw (CS), beet residues after extracting sugar (BR), and their blends, coal–corn straw blends (CCSBs), coal–wheat blends (CWBs), and coal–beet residues blends (CBRBs). The wheat, CS, and BR are three representative crops in the BM fractions. Different types of BM were observed in this study to give users more flexibility for choosing fuels, as different BMs have different harvest times. All CBBs had mass fractions of 50% coal and 50% BM.

This research aims to compare the thermal characteristics and kinetic behaviors of the aforementioned materials using a thermogravimetric (TG) analyzer to investigate the devolatilization and combustion characteristics under different heating rates, which were 10, 20, 30, 40, and 50 °C/min. Two representative kinetic methods were used to analyze the TG and differential thermogravimetric (DTG) results—namely, the Flynn–Wall–Ozawa (FWO) method and the Kissinger–Akahira–Sunose (KAS) method. The authors compared the combustion performances among different materials, in terms of ignition temperature (*T_i_*), burnout temperature (*T_b_*), and the combustion temperature (*T*_max_) at which the maximum mass loss rate (*dα/dt*)_max_ occurs. The calculation results regarding the activation energy (*E*) and preexponential factor (*A*) are presented as well. A comprehensive understanding of the combustion behavior of coal, BM, and CBB could provide guidelines for future biomass utilization, especially in the co-combustion process, based on this study. 

## 2. Materials and Methods

### 2.1. Materials Analysis

Coal was collected from the Maasvlakte energy plant in Rotterdam, The Netherlands. CS, wheat, and BR were collected from a Dutch farm. Before the TGA test, the materials were dried in an oven at 55 °C for two days and then ground into very small particles, with a diameter between 0.10 and 0.15 mm. Ultimate analyses for main elements (C, H, S, and N), lower heating value (LHV), and higher heating value (HHV) were tested by an organic elemental analyzer (ThermoFisher FLASH 2000), and the content of O was estimated by taking the complement. Proximate analysis was carried out using a TGA system (TA Instruments Q500). The proximate analysis of coal was based on the micro-TG method [31]. The biomass proximate method also used TGA [32]. The results of the ultimate and proximate analysis of samples of the basic ingredients are shown in Table 1.

### 2.2. Thermogravimetric Experiments

Gasification, pyrolysis, and combustion are the three major thermochemical conversion methods for transforming biomass and coal to energy [33]. A TA Instruments Q500 TGA was applied for the kinetic analysis of the thermal decomposition process. TGA can present a continued measurement of weight as a function of temperature. The initial sample masses were close to 10 mg. Three different CBBs were used in this study, which were coal–corn straw blends (CCSBs), coal–wheat blends (CWBs), and coal–beet residues blends (CBRBs). All CBBs had mass fractions of 50% coal and 50% BM. Dry air was used as the carrier gas. The heat balance was set automatically. The total gas flow rate was 50 mL min^−1^. Dynamic runs were performed at five different heating rates, which were 10, 20, 30, 40, and 50 °C min^−1^, from 30 °C to 1000 °C. The weight loss of seven samples at five heating rates was analyzed by TG and DTG data.

### 2.3. Kinetic Analysis

Kinetic analysis is a professional basis to research the parameters for the combustion process. The quantitative analysis could be applied to TGA results to obtain kinetic parameters of the thermal decomposition processes [34]. As regards the basic measurements, the parameter *r_m_* is the ratio of the mass; m represents mass; rm(t)=m(t)m0. Two universal kinetic expressions, Equations (1) and (2), for the entire reaction rate in gas–solid reactions are shown as follows, with α(t) being a nondimensional mass conversion ratio as function of time [22]:

(1) $$\alpha \left(t\right)=\frac{m_{0}-m\left(t\right)\;}{m_{0}-m_{f}}$$

where $m_{0}$ is the initial mass of the sample; m(t) is the mass of the sample at time *t*; $m_{f}$ is the final mass of the sample. The rate of change of *α* is given by the following equation:

(2) $$\frac{d\alpha }{dt}=kf\left(\alpha \right)$$

where *k* is the rate constant, and the differential reaction mechanism function is *f*(*α*). *f*(*α*) shows the changes in chemical or physical characteristics of the material during the process of reaction; it is defined by the reaction mechanism [14]. *k* is the combustion reaction rate, which is affected by the influence of the reactive gas partial pressure and temperature T. *k* is defined by the Arrhenius equation as follows:

(3) $$k=Ae^{\frac{-E}{RT}}$$

where the gas constant is *R* (8.314 J·K^−1^·mol^−1^), activation energy is *E* (kJ/mol), and the pre-exponential factor is *A* (min^−1^). Therefore, the principal Equation (4) of analytical methods to calculate kinetic parameters is obtained based on TGA results as follows:

(4) $$\frac{d\alpha }{dt}=Ae^{\frac{-E}{RT}}f\left(\alpha \right)$$

where the integral function G(α) can be obtained after integrating Equation (4), as follows:

(5) $$G\left(\alpha \right)=\int_{0}^{\alpha }\frac{d\alpha }{f\left(\alpha \right)}=A\int_{t_{0}}^{t}e^{\frac{-E}{RT}}dt$$

where a constant heating rate β is defined as $\beta =\frac{dT}{dt}$; Equation (5) can be transferred to [35] the following:

(6) $$G\left(\alpha \right)=\int_{0}^{\alpha }\frac{d\alpha }{f\left(\alpha \right)}=\frac{A}{\beta }\int_{T_{0}}^{T}e^{\frac{-E}{RT}}dT$$

Depending on the differential or integral form of Equation (4), various methods can be used to obtain the kinetic parameters *A* and *E* [36].

#### 2.3.1. Flynn–Wall–Ozawa Method

The authors used two different model-free methods in this work. The first one is the Flynn–Wall–Ozawa (FWO) method. Doyle’s approximation for the integral function was used for an accurate but easy and needed expansion (responsible for the suddenly appearing constants).

Writing the reaction rate in logarithmic form, Equation (6) is presented as follows [37,38]:

(7) $$\log\beta =\log\left(\frac{AE}{RG\left(\alpha \right)}\right)-2.315-0.4567\frac{E}{RT}$$

At a certain degree of conversion, *α*, the activation energy, *E*, is obtained for the set of multiple values of the heating rate, *β*, from the linear correlation of log*β* vs. 1/*T* because the logarithm at the right-hand side is constant as well in this case. Therefore, a straight line fitted through these points, which then has a slope of $-0.4567\frac{E}{RT}$. 

The estimation of the activation energy was based on this straight line fit. Based on the authors’ estimation, this fit was used according to the linear least-squares method. Therefore, the Pearson correlation coefficient between the individual points and the linear approximation at these locations could be applied to estimate the accuracy of the method. The pre-exponential factor, *A*, was evaluated from the intercept of $\log\left(\frac{AE}{RG\left(\alpha \right)}\right)-2.315$.

#### 2.3.2. Kissinger–Akahira–Sunose Method

The second model-free method used in this work is Kissinger–Akahira–Sunose (KAS) method. The principle of the KAS method is similar to the FWO method. This model is based on the following equation:

(8) $$\ln\left(\frac{\beta }{T^{2}}\right)=\ln\left(\frac{AR}{EG\left(\alpha \right)}\right)-\frac{E}{RT}$$

Then, for KAS as well, according to the linear relationship of $\ln\left(\frac{\beta }{T^{2}}\right)$ and $\frac{1}{T}$, a straight line could be obtained from the experimental data to calculate *E* of any conversion rate. The difference between the FWO method and the KAS method is the value of the left-hand side. For the FWO method, its left-hand side is a fixed value under the same heating rate. For the KAS method, it depends on the temperature as well. 

## 3. Results and Discussion

### 3.1. TG and DTG Analysis

#### 3.1.1. Combustion Characteristics of Each Sample

From Table 1, it can be observed that CS and wheat have 0% nitrogen and sulfur contents, the lowest level of ash, the highest level of volatile content, and a high value of HHV and LHV. This is an indication that CS and wheat are good options in BM combustion. In general, when comparing BMs with coal, it can be inferred that all C, N, and S contents in BMs are lower than that in coal, which means all BMs are better than coal. CS has the highest ratio of volatile to fixed carbon, demonstrating the easiest ignition level [14]. BR has lower values of volatile matter, HHV, and LHV, and a higher value of moisture, compared with CS and wheat, which shows that BR is a lower-grade biomass fuel, compared with CS and wheat. The descending sequence of fixed carbon concentration of all samples is coal > wheat > BR > CS. The sequence of HHV and LHV is coal > wheat > CS > BR. 

**Figure 1:**
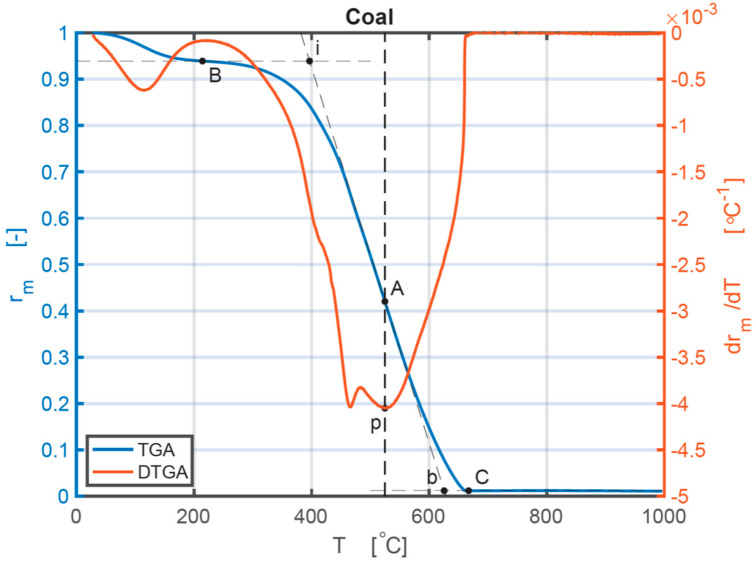
TG and DTG curves of the coal sample under 10 °C/min.

Coal was used to illustrate the analysis of combustion performances of the individual sample depending on TG and DTG curves. Figure 1 is a synoptical indication representing individual combustion stages under a heating rate of 10 °C min^−1^. The combustion stages considered are four important points that occur. The first one is the ignition point, *i*. The second one is the burnout point, *b*. The third one is the maximum mass loss rate point of the combustion phase, *p*. The fourth one is the point of combustion temperature (*T*_max_) at which the maximum mass loss rate (*dα/dt*)_max_ occurs. The intersection point of the vertical line that passes the point *p* and the TG curve is point *A*. The origin point of the thermal decomposition process after the water evaporation is point *B*. Point *C* is the point of completion from which the TG curve stabilizes to ashes. On the TG curve, a tangent at point A can be obtained. Through point *B*, a horizontal line is drawn. The ignition temperature, *T_i_*, is the interrelated temperature at the intersection of the horizontal line and the tangent at point *A* [39]. *T_i_* shows the ignition temperature of the sample and the difficulty level of the material to initiate combustion [40]. The burnout temperature, *T_b_*, is the corresponding temperature at the intersection of the tangent at *A* and the horizontal line through *C*. *T_i_*, *T_b_*, and *T*_max_ are all shown in Figure 1. It also displays each individual combustion process for coal. The complete thermal decomposition procedure could be separated into water loss (20 °C–200 °C), transition (200 °C–*T_i_*), combustion of volatile matter and fixed carbon (*T_i_*–*T_b_*), and burnout (*T_b_*–657 °C) sections. The *T_i_* temperature reflects how easily the ignition occurs. A lower *T_i_* value means an easier ignition start. The (*dα/dt*)_max_ and *T*_max_ indicate consecutive combustion and burning performances of the samples. The higher the value of (*dα/dt*)_max_ is, the higher the burning speed is after ignition. The combustion characteristics of four individual samples and three different CBBs are shown in Table 2. Coal has a higher *T*_max_, *T_i,_* and *T_b_* than the biomass samples at every heating rate. Therefore, biomass starts burning earlier, reaches the maximum mass loss point earlier, and finishes the burning earlier. For all three biomass samples, their *T_i_*, and *T*_max_ both increase with the grown of heating rates, which indicates BM usually burns quickly at a lower heating rate. The heating rate of 10 °C/min of BM has the lowest value of *T_i_,* which means a lower heating rate is appropriate for BM to start combustion. The combustion performance of coal does not show a consistent phenomenon with the increase in heating rates. Under the heating rate of 20 °C/min, the coal has the lowest *T*_i_ and *T*_max_, and the highest *T_b_*, which has the longest thermal reaction time. Based on the comparison of different kinds of BM, CS has the smallest value of *T_i_*, *T_b_*, and *T*_max_, while BR has the largest value of *T_b_* (the heating rates of 40 °C/min and 50 °C/min are not explicitly given in graphs or tables).

**Figure 2:**
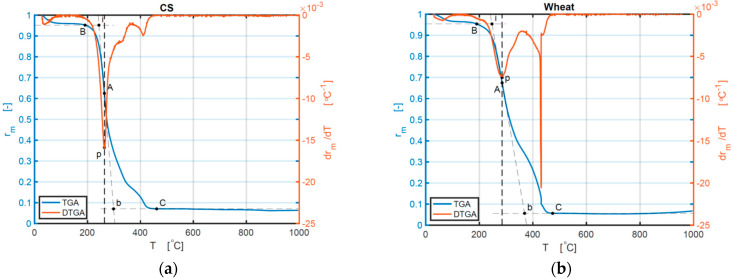

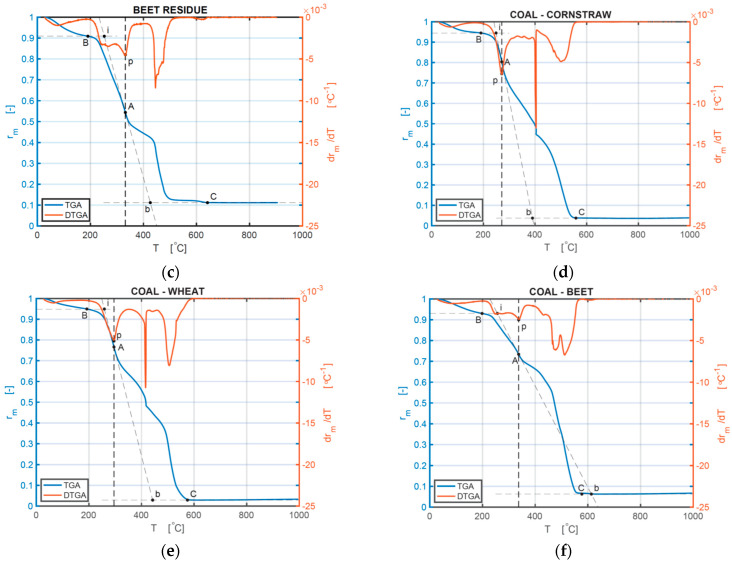
(**a**–**f**) TG and DTG curves of the samples of CS, wheat, BR, CCSB, CWB, and CBRB 10 °C/min: (**a**) TG and DTG curves of CS; (**b**) TG and DTG curves of wheat; (**c**) TG and DTG curves of BR; (**d**) TG and DTG curves of CCSB; (**e**) TG and DTG curves of CWB; (**f**) TG and DTG curves of CBRB.

The TG and DTG curves of other samples are shown in Figure 2a–f.

The mixture of biomass and coal has some special combustion performance, as indicated by the comparison of biomass and coal materials separately. The *T_i_* and *T*_max_ of CCSB only slightly increase, but *T_b_* increases significantly, compared with pure CS, which proves that adding coal powder can extend the combustion reaction period of CS but does not have a considerable influence on the ignition time and temperature of CS. For CWB, only at 10 °C/min do all *T_i_*, *T*_max_, and *T_b_* increase relative to pure wheat. For CBRB, there are no considerable differences in *T_i_* and *T*_max_ under all these heating rates (10, 20, and 30 °C/min), but there is a rather large difference in *T_b_*, compared with pure BR. Increasing the heating rates marginally improves *T_i_* and *T*_max_ for CCSB. The values of (−(*dα/dt*)_max_) are all decreased for CBB relative to BM, which means adding coal can slow down the combustion speed of BM. The ash fraction for all samples does not show a change with changing heating rates, but it shows a change between BM and CBB. For CS, the ash fraction decreases, compared with CCSB under 10 °C/min. For wheat and BR, the ash fraction decrease under all heating rates, in contrast to pure wheat and pure BR, which have a nonmonotonic behavior for these remains. The value of the ash fraction proves that adding coal powder to BM makes a more complete reaction process.

#### 3.1.2. Comparison between Coal, Biomass Materials, and Coal–Biomass Blends

**Figure 3:**
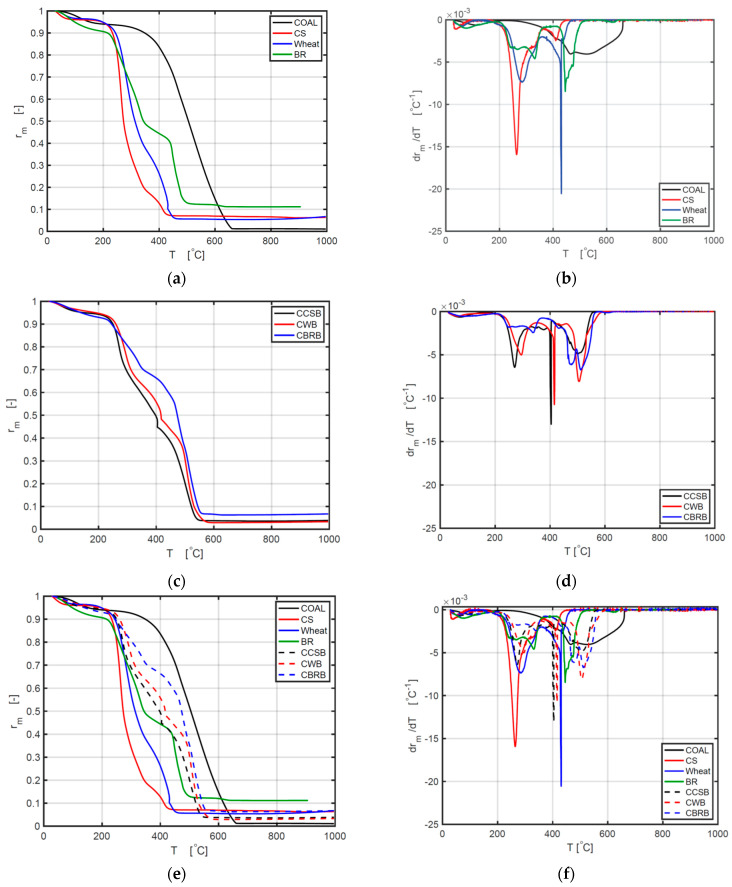
Comparative curves of TG and DTG between different samples under 10 °C/min: (**a**) comparison of TG curves between coal and biomass; (**b**) comparison of DTG curves between coal and biomass; (**c**) comparison of TG curves between different CBBs; (**d**) comparison of DTG curves between different CBB; (**e**) comparison of TG curves between all samples; (**f**) comparison of DTG curves between all samples.

Figure 3a shows a distinction in thermal characteristics of coal, CS, wheat, and BR. The same moisture dehydration scope could be found at about 50–200 °C. The sequence of weight loss between 50 °C and 200 °C is BR > coal > wheat > CS. This is consistent with the moisture contents of samples in Table 1. Figure 3c shows a similar process among thermal behaviors of CCSB, CWB, and CBRB, which is around 7%. 

As indicated by the curves of different BMs, they have similar ash fractions as follows:

For 10 °C/min, BR > CS > wheat;
For 20 °C/min and 30 °C/min, BR > wheat > CS.

The curves of different CBBs indicate the following ash fractions:

For 10 °C/min and 20 °C/min, CBRB > CCSB > CWB;
For 30 °C/min, CBRB > CCSB = CWB.

The weight loss rate ranking is CS > wheat > BR under all heating rates. 

The initial weight loss rate ranking is CCSB > CWB > CBRB for all heating rates. 

Therefore, adding coal powder into the biomass can slow down the burning velocity of every BM and can also increase the remaining ash fraction. Each DTG curve exhibits multiple dips (the term for the negative equivalent of a peak point on a graph) in the major thermal degradation phases, reporting the change in the reaction rates in the process of burning. The first very small dip value occurs in the temperature scope of 50−200 °C, while the second dip appears at 250−350 °C for BM and 420–530 °C for coal, and a third dip occurs at 400−500 °C for BM and 500–550 °C for coal. Hemicellulose and cellulose are the main chemical constituents in BM, and their thermal decomposition temperature scopes are from 200 to 320 °C, and from 320 to 400 °C, relatively, which is consistent with the combustion curve in Figure 3b. Figure 3b demonstrates that the burning rate of BM is distinctly higher than that of coal in the first and second processes, but the burning rate of coal is highest at the third stage. This is because the polymers of cellulose, lignin, and hemicelluloses are associated with comparably fragile bonds (380–420 kJ/mol energy), which are simple to be resolved [41,42]. Figure 3d shows DTG curves of different blends, which display four stages of mass losses. Stage 1 is the dehydration process, stage 2 is the release and combustion of volatile compounds, and stage 3 belongs to the release and combustion of char oxidation. Stage 4 occurs mostly because of coal combustion [14]. After adding coal powder, CCSB, and CWB have a higher combustion rate at the second and third stages.

From Figure 3e, it can be concluded that all CBBs showed a higher reaction rate and a higher mass conversion rate, compared with single BM. The values of *T_i_*, *T_b_*, *T*_max_, and −(*dα/dt*)_max_ of CBB are all between the values of BM and coal. These results showed more specific details as to how coal and biomass affect each other. As previous researchers have only concluded, there might be a synergistic interaction between biomass and coal. Through Figure 3f, the reaction rates of CBB are slower than BM on the second and third stages, compared with BM. Figure 3f provides detailed information on how the reaction rate is during the whole reaction process. 

### 3.2. Analysis of Two Kinetic Methods

#### Calculated Kinetic Parameters

**Figure 4:**
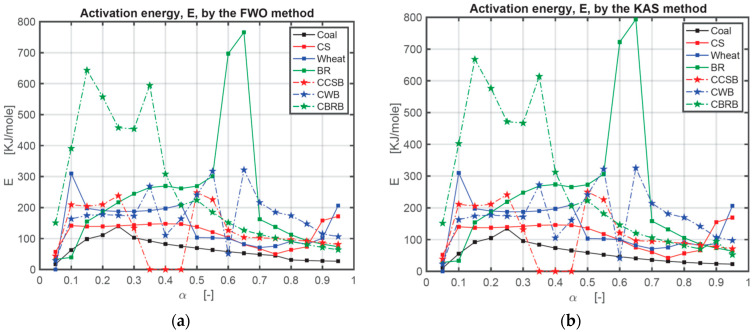
Comparison of activation energy (*E*) obtained with FWO and KAS method: (**a**) comparison of activation energy (*E*) obtained with FWO method; (**b**) comparison of activation energy (*E*) obtained with KAS method.

**Figure 5:**
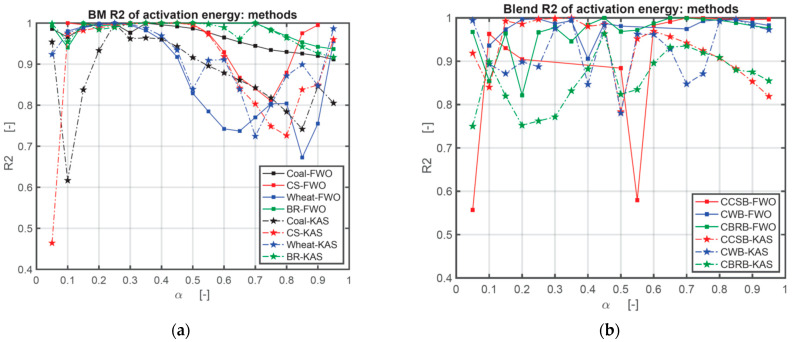
Comparison of correlation coefficient (*R*^2^) obtained with FWO and KAS methods: (**a**) comparison of correlation coefficient (*R*^2^) of coal and BM obtained with FWO and KAS methods; (**b**) comparison of correlation coefficient (*R*^2^) of CBB obtained with FWO and KAS methods.

The activation energy (*E*) obtained by the FWO method and KAS method are listed in numerical form in Table A1 in Appendix A and graphically in Figure 4. In this research, the authors used three heating rates series: 10, 20, and 30 °C/min; 10, 30, and 50 °C/min; 10, 20, 30, 40, and 50 °C/min. As the sensitivity of the TG setup is relatively high, the authors recorded the *E* value under the highest *R*^2^ in the case of all positive numbers. There are still some negative numbers after all calculations. In Table A1, * means the results calculated under the heating rates series of 10, 20, 30 °C/min; ** means the results calculated under the heating rates series of 10, 30, 50 °C/min; *** means the results calculated under the heating rates series of 10, 20, 30, 40, 50 °C/min. At the same conversion level (*α*), the plot of log *β* against 1/*T* indicates a linear relation with the various heating rates for the FWO method [43], and the plot of ln (*β*/*T*^2^) against 1/*T* indicates a linear relation with the various heating rates as well for the KAS method. The activation energy (*E*(*α*)) varies with the mass conversion degree (*α*) [44,45]. The deviation could be as large as around 25%, so the deviation below 20% of the FWO method is appropriate [46]. Figure 4 is the comparison of *E* obtained with the FWO method and KAS method depending on the results from Table A1 in Appendix A. Figure 5 is the comparison of the correlation coefficient (*R*^2^) obtained with the FWO and KAS methods. Figure 5 shows that the *R*^2^ of the FWO method is closer to 1.00 and more stable for separate samples and mixed samples. Therefore, the authors used the FWO method to calculate another kinetic parameter, the pre-exponential factor, *A*.

From the activation energy results, coal shows the lowest value of *E*, and BR shows the highest value of *E*. For coal and BR only, these values increase first and then decrease, which means the reaction happens easily at the beginning and ending phases. For coal, when 15% < *α* < 35%, the activation energy *E* is the highest, and when 80% < *α* < 95%, it is the lowest. The longer the reaction takes, the easier the reaction proceeds. Corn straw, CS, shows relatively stable *E* values between 10% and 55%, which means a stable reaction degree. When 70% < *α* < 85%, the activation energy is the lowest. Wheat results show a similar stable phenomenon as CS between 15% and 45% and have the lowest *E* value between 65% and 90%. In the beet residue case, BR, the reaction process is more complicated. When 25% < *α* < 50%, it has a stable reaction, and then when 60% < *α* < 65%, it becomes difficult suddenly. All CBBs have four phases, classified, respectively, into difficult reaction, easy reaction, difficult reaction again, and easy reaction again. When 5% < *α* < 25%, the order of *E* is *E*_Wheat_ > *E*_BR_ > *E*_CS_ > *E*_Coal_, which means at the beginning period of combustion, wheat needs the most energy to react. For 25% < *α* < 45%, the order of *E* is *E*_BR_ > *E*_Wheat_ > *E*_CS_ > *E*_Coal_, which means at this stage, BR is the most difficult to burn. For a conversion ratio 45% < *α* < 60%, the order of *E* is *E*_BR_ > *E*_CS_ > *E*_Wheat_ > *E*_Coal_. When the 60% < *α* < 85%, the order of *E* is *E*_BR_ > *E*_Wheat_ > *E*_CS_ > *E*_Coal_. Therefore, in this study, the conclusion can be obtained that coal is much easier to combust than BM, while CS is relatively the easiest BM for combustion in this research, compared with other BMs. Adding coal to CS does not have a significant effect on its reaction. For wheat, adding coal improves its combustion speed at the beginning when *α* < 45%. Adding coal to BR makes the reaction easier when *α* > 45%. Therefore, adding coal to BMs can help improve their combustion properties. 

## 4. Conclusions

The work presented studied the effect of the co-combustion of coal and biomass samples on combustion characteristics and kinetic parameters. Combustion of coal, three types of biomass (BM), and three kinds of coal biomass blends (CBBs) were investigated using a thermogravimetric analyzer. Through this research, corn straw (CS) and wheat show better combustion properties than beet residue (BR). Therefore, they are recommended to be used in biomass combustion. The heating rate of 10 °C/min of BM has the lowest value of the ignition temperature, (*T_i_*), which means a lower heating rate is appropriate for BM to start combustion. When the heating rate is 20 °C/min, coal was observed to have the longest thermal reaction time when compared with 10 and 30 °C/min. The ignition temperature (*T_i_*) and the maximum mass loss temperature (*T*_max_) of the coal corn straw blend (CCSB) only slightly increase, but the burnout temperature (*T_b_*) increases significantly, as opposed to pure corn. In corn straw (CS), it is proven that adding coal powder can extend the combustion reaction period of CS. In the case of blending wheat with coal, CWB, only under 10 °C/min, all *T_i_*, *T*_max_, and T_b_ increase, compared with pure wheat. Mass losses (−(*dα/dt*)_max_) decreased for CBB, compared with BM.

For the combustion performance, the conclusion is that adding coal to the biomass improves the burnout level, slows down the burning speed, and increases the overall combustion reaction time. Equating the analysis methods FWO and KAS, it is found that, in the current case, the FWO analysis method is the best method to calculate the kinetic parameters for coal, BM, and CBB. FWO analysis shows good accuracy as derived from higher observed correlation values, R. In the case of coal, when 15% < *α* < 35%, the activation energy *E* is the highest, but when 80% < *α* < 95%, the E is the lowest. The longer the reaction takes, the easier the reaction proceeds. 

Adding coal to wheat and BR can change their activation energy, according to this study, making it lower and more stable under some specific *α*. Therefore, adding coal to biomass helps improve their combustion properties. The conclusions of this article are consistent with results from previous researchers indicating there might be a synergistic interaction between biomass and coal. The thermal reactivity and ignition performance will be upgraded by burning the biomass and coal together. It is insightful to research the co-combustion of biomass and coal. Blending with coal is a good means of transition for biomass combustion from the perspectives of combustion performances and kinetics analysis. The authors selected a wider range of biomass raw materials, made more kinds of CBB, and conducted more studies on different heating rates. According to the data in this article, users of thermal power plants can select different types of BM and coal for co-combustion in different seasons. According to the combustion characteristics and kinetics parameters of the CBBs found in this article, the operator of the thermal power plant will have a better understanding of how to operate the machine and control the boiler.

## Figures and Tables

**Table 1:** Proximate analysis, ultimate analysis, and thermal properties of the unblended samples.

Characteristic	Coal	Corn Straw	Wheat	Beet Residues
Proximate analysis (wt %)				
Moisture	6.61	5.50	5.81	9.95
Ash	37.00	7.47	4.99	27.28
Volatile matter	3.72	69.18	54.45	43.13
Fixed carbon	52.67	17.85	34.75	19.64
Ultimate analysis (wt %, daf)				
C	52.19	33.09	32.85	29.68
H	3.24	3.94	4.01	3.96
S	0.36	0	0	0.23
N	0	0	0	0
O ^a^	44.21	62.97	63.14	66.13
HHV (kcal/kg)	5404	4070	4073	3801
LHV (kcal/kg)	5238	3868	3868	3598

^a^ Estimated by difference. daf: dry ash-free.

**Table 2:** *T_i_*, *T_b_*, and *T*_max_ of all samples.

Sample	*T_i_* (°C)	*T*_max_ (°C)	*T_b_* (°C)	Ash Fraction	*−(dα/dt)*_max_
**10 °C/min**					
Coal	397	525	626	0.12	4.0 × 10^−3^
CS	243	264	299	0.07	15.9 × 10^−3^
Wheat	247	285	369	0.06	74 × 10^−3^
BR	252	332	426	0.11	4.6 × 10^−3^
CCSB	249	271	390	0.04	6.4 × 10^−3^
CWB	259	295	443	0.03	5.0 × 10^−3^
CBRB	257	337	613	0.06	2.4 × 10^−3^
**20 °C/min**					
Coal	383	435	808	0.17	1.80 × 10^−3^
CS	251	275	312	0.03	15.8 × 10^−3^
Wheat	261	299	364	0.07	8.6 × 10^−3^
BR	254	338	438	0.11	4.4 × 10^−3^
CCSB	260	281	367	0.03	8.7 × 10^−3^
CWB	256	299	483	0.02	4.0 × 10^−3^
CBRB	268	346	591	0.07	2.7 × 10^−3^
**30 °C/min**					
Coal	409	450	700	0.07	3.0 × 10^−3^
CS	260	279	317	0.03	16.5 × 10^−3^
Wheat	266	309	375	0.06	8.2 × 10^−3^
BR	263	343	436	0.09	4.8 × 10^−3^
CCSB	269	290	335	0.03	14.0 × 10^−3^
CWB	275	313	356	0.03	5.1 × 10^−3^
CBRB	264	346	622	0.06	2.4 × 10^−3^

## Data Availability

No appliable.

## Abbreviations

BM	Biomass materials
TGA	Thermogravimetric analysis
CBBs	Coal–biomass blends
CS	Corn straw
BR	Beet residues after extracting sugar
CCSBs	Coal–corn straw blends
CWBs	Coal–wheat blends
CBRBs	Coal–beet residues blends
HHV	Higher heating value
LHV	Lower heating value

## Appendix A

**Table A1:** Activation energy and its correlation accuracy.

**FWO**			**KAS**		**FWO**		**KAS**		**FWO**		**KAS**	
**α**	***E*_Coal_** **KJ/mol**	***R* ^2^_Coal_**	***E*_Coal_** **KJ/mol**	***R* ^2^_Coal_**	***E*_CS_** **KJ/mol**	***R* ^2^_CS_**	***E*_CS_** **KJ/mol**	***R* ^2^_CS_**	***E*_Wheat_** **KJ/mol**	***R* ^2^_Wheat_**	***E*_Wheat_** **KJ/mol**	***R* ^2^_Wheat_**
5	17.42	0.9860 *	10.39	0.9470 *	56.99	0.2863 ***	52.04	0.2321 ***	NVE	NVE	NVE	NVE
10	62.97	0.9658 **	55.29	0.9522 **	141.63	1.0000 *	140.43	1.0000 *	310.00	0.9804 **	317.37	0.9793 **
15	98.48	0.9889 **	92.06	0.9863 **	139.14	0.9961 *	137.61	0.9956 *	197.61	0.9914 *	198.91	0.9906 *
20	110.97	0.9979 **	104.77	0.9973 **	139.05	0.9962 *	137.40	0.9957 *	189.90	0.9984 *	190.64	0.9983 *
25	139.50	0.9987 *	134.63	0.9984 *	141.00	0.9978 *	139.37	0.9974 *	187.60	0.9998 *	188.10	0.9997 *
30	102.76	0.9764 **	95.42	0.9694 **	143.57	0.9991 *	142.02	0.9990 *	187.62	0.9950 *	188.01	0.9945 *
35	92.28	0.9995 **	84.11	0.9992 **	146.59	0.9999 *	145.14	0.9999 *	190.36	0.9822 *	190.77	0.9804 *
40	82.26	0.9959 **	73.29	0.9946 **	147.72	1.0000 *	146.28	1.0000 *	196.97	0.9593 *	197.61	0.9554 *
45	75.39	0.9919 **	65.77	0.9888 **	146.89	1.0000 *	145.35	1.0000 *	208.94	0.9173 *	210.10	0.9102 *
50	69.20	0.9869 **	58.94	0.9810 **	137.70	0.9967 *	135.62	0.9962 *	103.34	0.8290 **	223.36	0.8012 *
55	63.21	0.9769 **	52.32	0.9645 **	120.78	0.9754 *	117.72	0.9714 *	102.48	0.7847 **	97.62	0.7490 **
60	57.70	0.9649 **	46.20	0.9424 **	103.42	0.9293 *	99.33	0.9161 *	100.39	0.7424 **	95.22	0.7006 **
65	52.81	0.9536 **	40.70	0.9187 **	80.62	0.8670 *	75.15	0.8362 *	82.17	0.7370 **	75.70	0.6820 **
70	48.59	0.9443 **	35.88	0.8951 **	67.19	0.8402 *	60.79	0.7948 *	70.98	0.7703 **	63.43	0.7066 **
75	45.05	0.9346 **	31.77	0.8673 **	50.03	0.8104 *	42.39	0.7338 *	75.55	0.8034 **	67.87	0.7477 **
80	30.97	0.9298 *	28.50	0.8370 **	63.88	0.8792 **	56.67	0.8380 **	90.96	0.8042 **	83.88	0.7587 **
85	29.18	0.9258 *	25.95	0.8064 **	73.28	0.9752 **	66.30	0.9671 **	81.47	0.6726 ***	73.80	0.6036 ***
90	27.84	0.9202 *	24.02	0.7762 **	158.10	0.9952 **	155.12	0.9944 **	101.16	0.7551 **	94.28	0.7072 **
95	26.89	0.9117 *	22.75	0.7475 **	171.75	0.2295 **	169.23	0.2073 **	206.35	0.9560 *	205.10	0.9509 *
**FWO**			**KAS**		**FWO**		**KAS**		**FWO**		**KAS**	
***α***	***E*_BR_** **KJ/mol**	***R* ^2^_BR_**	***E*_BR_** **KJ/mol**	***R* ^2^_BR_**	***E*_CCSB_** **KJ/mol**	***R* ^2^_CCSB_**	***E*_CCSB_** **KJ/mol**	***R* ^2^_CCSB_**	***E*_CWB_** **KJ/mol**	***R* ^2^_CWB_**	***E*_CWB_** **KJ/mol**	***R* ^2^_CWB_**
5	32.93	0.9925 *	28.29	0.9893 *	43.73	0.5570 **	38.80	0.4711 **	29.24	0.2763 **	22.97	0.1761 **
10	39.15	0.9404 *	33.27	0.9123 *	209.31	0.9631 **	211.30	0.9600 **	163.60	0.9361 **	163.01	0.9292 **
15	154.77	0.9997 *	154.17	0.9997 *	203.99	0.9304 ***	205.46	0.9245 ***	174.62	0.9734 **	174.33	0.9704 **
20	185.29	0.9928 *	186.03	0.9921 *	210.00	0.9043 ***	211.65	0.8966 ***	177.59	0.9966 **	177.25	0.9962 **
25	216.89	0.9960 *	219.05	0.9957 *	238.23	0.7996 ***	241.22	0.7872 ***	174.74	0.9976 **	174.08	0.9973 **
30	244.52	0.9999 *	247.89	0.9999 *	133.76	0.1770 ***	131.24	0.1577 ***	172.78	0.9866 **	171.80	0.9849 **
35	264.74	0.9995 *	268.93	0.9995 *	NVE	NVE	NVE	NVE	269.01	0.9933 *	272.73	0.9928 *
40	269.51	0.9996 *	273.71	0.9996 *	NVE	NVE	NVE	NVE	110.75	0.9058 **	105.40	0.8867 **
45	261.82	0.9997 *	265.40	0.9997 *	NVE	NVE	NVE	NVE	164.00	0.9874 **	161.02	0.9855 **
50	269.09	0.9998 *	272.86	0.9997 *	248.23	0.8841 **	249.86	0.8747 **	240.90	0.9810 **	241.68	0.9791 **
55	300.94	0.9975 *	306.18	0.9974 *	225.86	0.5795 **	226.09	0.5553 **	317.59	0.8713 **	322.14	0.8629 **
60	696.87	0.1561 *	722.09	0.1523 *	126.65	0.9830 **	121.18	0.9797 **	51.13	0.1120 ***	41.65	0.0704 ***
65	765.87	0.9992 *	793.69	0.9992 *	103.87	0.9911 **	96.63	0.9889 **	321.70	0.7255 **	325.49	0.7099 **
70	162.12	1.0000 *	158.32	1.0000 *	102.35	0.9999 **	94.68	0.9999 **	216.28	0.9745 **	214.32	0.9712 **
75	137.47	0.9837 *	132.25	0.9804 *	100.48	0.9995 **	92.47	0.9993 **	185.12	0.9927 **	181.39	0.9915 **
80	112.25	0.9684 *	105.56	0.9604 *	96.72	0.9977 **	88.28	0.9968 **	173.98	0.9958 *	169.67	0.9950 *
85	93.28	0.9530 *	85.40	0.9382 *	91.73	0.9965 **	82.81	0.9949 **	147.54	0.9952 *	141.72	0.9944 *
90	82.05	0.9424 *	73.38	0.9211 *	86.69	0.9964 **	77.27	0.9945 **	115.17	0.9890 **	107.23	0.9856 **
95	73.62	0.9368 *	64.27	0.9096 *	81.27	0.9967 **	71.31	0.9948 **	106.20	0.9825 **	97.49	0.9766 **
**FWO**							**KAS**					
***α***	***E*_CBRB_** **KJ/mol**			***R* ^2^_CBRB_**			***E*_CBRB_** **KJ/mol**			***R* ^2^_CBRB_**		
5	150.76			0.9676 *			151.71			0.9648 *		
10	390.99			0.8539 *			402.56			0.8485 *		
15	643.46			0.9648 *			667.61			0.9639 *		
20	557.29			0.8214 *			576.54			0.8166 *		
25	457.81			0.9666 *			471.47			0.9652 *		
30	453.86			0.9776 *			466.96			0.9766 *		
35	594.38			0.9458 **			613.98			0.9439 **		
40	308.17			0.9843 **			312.25			0.9831 **		
45	207.35			0.9998 *			205.80			0.9998 *		
50	223.80			0.9686 **			222.76			0.9650 **		
55	185.11			0.9717 **			181.89			0.9675 **		
60	151.54			0.9872 **			146.38			0.9847 **		
65	126.87			0.9998 **			120.22			0.9997 **		
70	113.52			0.9995 **			105.92			0.9993 **		
75	101.45			0.9970 **			93.01			0.9958 **		
80	90.65			0.9944 **			81.44			0.9918 **		
85	80.38			0.9887 **			70.39			0.9828 **		
90	71.41			0.9819 **			60.66			0.9707 **		
95	63.79			0.9766 **			52.32			0.9595 **		

* Calculated under the heating rates series of 10, 20, 30 °C/min; ** calculated under the heating rates series of 10, 30, 50 °C/min; *** calculated under the heating rates series of 10, 20, 30, 40, 50 °C/min; NVE: no value, because the sample weight is minuscule, and the machine is so sensitive, there is no specific value after calculation. This can exist in the experiment. This only appears at a few *α*.
